# Obtaining Consent for Future Research with Induced Pluripotent Cells: Opportunities and Challenges

**DOI:** 10.1371/journal.pbio.1000042

**Published:** 2009-02-24

**Authors:** Katriina Aalto-Setälä, Bruce R Conklin, Bernard Lo

## Abstract

Human induced pluripotent stem cells have revolutionized stem cell research. However, their great potential also raises important concerns about consent for future research.

The recent development of human induced pluripotent stem (iPS) cells [1–5] has reshaped the scientific and political landscape of stem cell biology. iPS cells provide an unprecedented opportunity to study the pathophysiology of diseases, understand stem cell biology, identify new therapeutic targets, and test new therapies. Furthermore, they offer the possibility of transplanting therapeutic cells that are genetically identical to their recipient.

iPS cells are not included in the heated debates over the ethics of embryonic stem cell research because embryos or oocytes are not used. The President's Council on Bioethics called iPS cells “ethically unproblematic and acceptable for use in humans” [6]. Currently, there are no restrictions on federal funding of iPS cell research, and iPS cells are not subject to the special regulations in place for embryonic stem cells [7]. While neither the donation of materials to derive iPS cells nor their derivation raises special ethical issues, some potential downstream uses of iPS derivatives may be so sensitive as to call into question whether the original somatic cell donors would have agreed to such uses. In light of the enormous scientific and public interest in iPS cells and claims about their lack of ethical problems, it is important to consider these downstream issues now. Although these concerns also apply to other types of stem cell and genomics research, they are particularly salient to iPS research for two reasons. First, if the perception that iPS research poses no ethical concerns is not corrected, there could be a backlash against iPS cells later. Second, the virtual genetic identity between iPS cells and donor cells raises particular concerns regarding respect for donors.

Because human biological materials are precious and iPS cells can be propagated indefinitely, it is advisable to design the consent process for donating somatic cells for iPS derivation to facilitate a broad range of future research, beyond what the initial investigators may have in mind. The US National Academy of Sciences recently revised its guidelines for stem cell research [7]. In this paper, we go beyond the National Academy of Sciences report to recommend how the consent process for the donation of somatic cells to derive iPS cell lines should foster their future scientific uses, particularly in fundamental research to study the properties of stem cells, derive specialized cells, and carry out preclinical studies of transplantation.

## Consent for Derivation of Human iPS Cells

The research groups that first reported the derivation of human iPS cells used commercially available “de-identified” human somatic cells [2–4,8]. Using previously collected cells may be advantageous if they are well characterized and scientists have extensive experience growing them and working with them.

Generally, informed and voluntary consent in research is required to fulfill the ethical principle of respect for persons. This requirement is identified as fundamental in international standards, such as the Helsinki Declaration and the Council for International Organizations of Medical Sciences' “International Ethical Guidelines for Biomedical Research Involving Human Subjects,” in good clinical practice standards, in US regulations for the protection of human research participants [9–14], and in stem-cell-specific standards from the International Society for Stem Cell Research, the US National Academy of Sciences, and the California Institute for Regenerative Medicine [15–17].

Under US regulations for research on humans, an exception to consent allows existing biological materials to be used for research without consent if they are de-identified. The rationale for this exception is that there is no risk of physical harm to donors, and de-identifying materials greatly reduces the risk of breaches of confidentiality. However, use of de-identified samples raises concerns about confidentiality and the possibility that donors might not approve of certain downstream research uses. Existing biological materials may also be used for research if the donor has consented, which may be simply a general consent to research. These policies regarding research with existing biological materials enjoy widespread but not unanimous public support. A 2006 paper reviewed 30 papers on attitudes toward consent for research with human biological samples [18]. Of 20 studies that surveyed willingness to donate tissues, 17 reported that over 80% of respondents said they would donate a sample if asked. Furthermore, in six studies that addressed the issue, 79%–95% of respondents were willing to provide one-time general consent and to allow ethics committees to determine the studies for which their samples would be used. None of the reviewed studies, however, addressed stem cell research or informed respondents about potentially sensitive research that might be carried out using their specimens.

A great advantage of iPS cells is that they can be derived from a person carrying a specific gene mutation or having a specific disease or condition, thereby creating an in vitro model for human diseases. Thus, many new iPS cell lines will be created from newly donated biological materials [8].

## Future Fundamental Research with iPS Cells

Wide sharing of iPS lines will facilitate progress in this exciting field. It will be impossible to predict whether an individual iPS line will be particularly well suited for specific functions, such as deriving therapeutic cell types or screening drug responses. Future researchers will likely want to carry out a broad range of basic experiments with iPS cells and derivatives, using common and well-accepted scientific practices (Box 1).

**Box 1. Future Basic Research Uses of iPS Cells**

- Genetic modification of cells
- Injection of iPS cells or derivatives into nonhuman animals, including injection into the brain
- Large-scale genome sequencing
- Sharing cell lines with other researchers, with appropriate confidentiality protections
- Patenting scientific discoveries and developing commercial tests and therapies, with no sharing of royalties with donors

These standard research techniques are widely used in other types of basic research and provide important scientific information. Large-scale genome sequencing is likely to yield new insights about the pathogenesis of disease and to identify new targets for therapy. Injection of human stem cells into the brains of nonhuman animals will be required for preclinical testing of cell-based therapies for many conditions, such as Parkinson disease, Alzheimer disease, and stroke. Generally, donors of biological materials are not explicitly informed of these research procedures, although such disclosure is now proposed for whole-genome sequencing [20,21].

However, this research could raise concerns. For example, large-scale genome sequencing may evoke concerns about privacy and confidentiality. Donors might consider their privacy violated if scientists know their future susceptibility to many genetic diseases. Furthermore, re-identification of the donor of a de-identified large-scale genome sequence might be possible if confidentiality is breached at forensic DNA databases or an Internet company offering personal genomic testing [22,23].

Other donors may object to their cells being injected into animals. For example, they may oppose all animal research, or they may have religious objections to the mixing of human and animal species. The injection of human neural progenitor cells into nonhuman animals has raised ethical concerns about animals developing characteristics considered uniquely human [24,25]. Transplanted cells are influenced by the microenvironment in the recipient [26,27], and human neural progenitor cells have been transplanted into developing animal brains without observed behavioral alterations [28]. However, we do not know exactly what behavioral components to look for or how to measure mental capacities in animals. The possibility of creating a more human-like animal may be greater if most of the animal brain cells at the early stage of development are replaced in a primate species closely related to humans.

We note that these ethical concerns are not unique to iPS research. They apply to other stem cell research and to large-scale genomic sequencing. These ethical concerns may be resolved if somatic cell donors give consent for derivatives of their cells to be used in such research. Later in the paper, we discuss how such consent for future research may be obtained.

## Future Sensitive Research with iPS Cells

Two types of future research with iPS cells—transplantation and reproductive research—are likely to be particularly controversial, both because many somatic cell donors can be predicted to disapprove of them and because explicit consent is generally required for these activities in other settings. Human transplantation of iPS cells is not feasible in humans with current techniques of inducing pluripotency that require integration of foreign DNA. However, it is widely anticipated that “integration-free” iPS cells will be developed in the next few years, making human transplantation possible.

### Transplantation into humans.

For transplantation of solid organs, tissues, or allogenic cord blood, expressed consent is required from donors or their surrogates. Transplantation of cells derived from iPS cells would differ from organ and tissue transplantation because obtaining tissue for iPS cells is minimally invasive. However, some people may not want their cells to become an integral, growing part of another person. While many people are eager to give the “gift of life,” others choose not to donate organs [29]. “Presumed” consent for cadaveric transplantation has scant support in the US [30]. Prior consent for unspecified research would not resolve the problem, because donors may not have considered the possibility of transplantation when providing somatic cells. De-identifying donor cells also does not eliminate their objections. Thus, respecting donor autonomy would require explicit consent for stem cell transplantation.

An additional reason for obtaining explicit consent for allogenic transplantation is the safety of transplant recipients. The interval between the original donation of the somatic cells and the proposed stem cell transplantation might be many years. If the donor developed cancer, or if a strong family history of cancer was recognized during this time, the risks to recipients might be increased. Screening the materials to be transplanted (e.g., for karyotype abnormalities or for alleles strongly associated with cancer) would fail to identify some risks to recipients, because the genetic basis of many inherited cancers has not been identified. The importance of careful screening of iPS cells is magnified by the fact that these cells can be propagated for many passages, and many patients may receive stem cell transplants from a single donor. Banks of just a few hundred carefully selected stem cells might serve the transplant needs of a large portion of the population. Thus, it would be desirable to periodically obtain an updated medical and family history from the donor of a somatic cell line that is being used for repeated transplantations. Recontacting donors without their prior permission, however, might be considered an invasion of privacy. Hence, consent for recontact should be obtained when the somatic cells are donated and approved by the institution's internal review board (IRB) or stem cell research oversight (SCRO) committee.

### Reproductive research.

Human iPS cells might be made to differentiate into primordial germ cells and then into mature gametes [31]. Gametes derived from iPS cells would be useful both for understanding gametogenesis and as a potential infertility treatment [31]. Gametes derived from iPS cells would have virtually the same DNA as the somatic cell donor.

Basic research on gamete maturation raises few ethical concerns. Studying how gametes develop is similar to studying how neural cells, cardiomyocytes, and beta islet cells develop. However, using gametes derived from iPS cells for research on reproduction may raise serious ethical objections because of the moral significance of reproduction and strong disagreements over the moral status of embryos [32–34]. Furthermore, if iPS cells are actually totipotent and could potentially be used for reproductive cloning, additional strong moral objections are likely to develop [32,35].

Many Americans—as many as 62% in some opinion polls—believe that embryos should not be created specifically for research purposes [36]. However, some somatic cell donors would welcome research to create embryos with gametes derived from iPS cells because it might lead to breakthroughs in infertility treatment. On the other hand, some people can be expected to object to using such gametes for reproductive research involving fertilization, parthenogenesis, or androgenesis. For example, some people strongly believe that embryos should only be created through sexual intercourse and that in vitro fertilization violates natural law and divine commandment [33]. These individuals would object to such reproductive research using gametes derived from their somatic cells, as well as carrying out reproductive research without specific consent from somatic cell donors. De-identifying the iPS derivatives would not overcome their opposition.

Thus, explicit, separate consent from donors of somatic cells should be required for reproductive research that attempts to create totipotent cells from gametes derived from iPS derivatives. In light of the objections raised to reproductive cloning [32,35], we believe that research for this purpose should be prohibited.

## Suggested Informed Consent Procedures

No one can predict if an iPS line being derived will become highly desirable for use in future research (e.g., because it grows robustly in culture). When researchers obtain somatic cells to derive new iPS lines, they should also ask donors for permission to carry out additional basic research that will be needed to reap the hoped-for scientific benefits of these remarkable cells. Flawed consent for the donation of materials to derive human stem cell lines has called into question downstream uses of the lines [7,37].

There are conflicting ethical and policy considerations regarding future research with biological materials. On the one hand, researchers must respect persons who donate materials [12,38,39]. However, it is difficult to specify what the level of detail in the consent process should be. Exhaustive disclosure of the details of research may be confusing and counterproductive. We contend that participants ought to appreciate essential features of the project, particularly those pertinent to decisions to participate or not participate. Unless they are told, donors might not realize that some research might be considered objectionable to a substantial minority of donors; large-scale genome sequencing has been cited as an example [40,41]. Future research presents additional challenges regarding consent. Blanket consent or general consent, in which donors agree to all unspecified future studies using their materials, may be uninformed if donors are not told about the kinds of projects that might be considered objectionable [42].

On the other hand, scientifically essential and ethically acceptable research should be encouraged for the benefit of society and future patients. Undue delays and administrative burdens on such research should be minimized. It would be inefficient for scientists to invest time and effort to derive a new iPS line with cells from a donor who would not agree to basic research procedures needed to make use of these valuable cell lines. For example, the scientific usefulness of a line would be compromised if the donor would not allow injection into animals or large-scale genome sequencing.

We recommend that scientists use somatic cells only from donors who agree to the basic research procedures listed in Box 1. During the consent process, researchers need to help donors understand that these techniques are essential to achieve the goals of understanding diseases and developing new therapies and that they are widely used in other types of research, such as cancer research. Donors should also be invited to ask questions. Although these research procedures should be discussed with the IRB or SCRO committee reviewing the protocol, they may not need to be discussed with donors of biological materials during the consent process. It would be administratively simpler if widely distributed cell lines could be used for all the procedures in Box 1, rather than imposing different restrictions on different lines. There is likely to be no shortage of donors for iPS research who agree to allow derivatives of their cells to be used in this range of fundamental research. In rare situations, it may be desirable to derive iPS lines from donors who do not agree to these conditions. For example, a donor with a rare condition or mutation that would be extremely valuable to study may not agree to large-scale genome sequencing or injection into animals, even after attempts to explain the importance of such downstream research.

As noted, concerns about consent for future research with biological materials can also be raised about other types of research, including research with other types of stem cells. Researchers may also wish to ask donors to agree to the procedures in Box 1, to facilitate future studies.

We further recommend obtaining permission to recontact donors in the future, subject to IRB approval. The ability to recontact donors offers the opportunity to discuss future research that is so innovative that it cannot be anticipated today [43], and that IRBs or SCRO committees might consider outside the scope of the original consent.

Clinical transplantation and reproductive research deserve special consideration. Some people who support the basic iPS research listed in Box 1 might object to these applications. However, there would be little lost in terms of fundamental stem cell biology if some somatic cell donors excluded their cells from transplantation or reproductive research. Thus, we suggest a tiered consent process. After obtaining consent for derivation and the procedures in Box 1, researchers should routinely ask for additional consent to use derived cells for clinical transplantation. We believe that permission for reproductive research should be sought separately and only in exceptional cases, when researchers can foresee colleagues carrying out such work, because some donors will have deeply rooted objections. Donors could still provide cells for iPS derivation and the research listed in Box 1, even if they decline these additional uses.

Our recommendations are intended as ethical guidelines, not as regulatory guidelines. Many aspects of human stem cell research are not, strictly speaking, research on humans. Many scientists and policy makers were reluctant to subject human embryonic stem cell research to the federal regulations for research on human participants. Difficult ethical issues were not covered by these regulations, and IRBs as currently constituted did not have the expertise to address stem-cell-specific issues [16]. Currently, iPS cell research may be subject to state stem cell research regulations. We believe that voluntary professional guidelines should play a crucial important role at this time. As we have argued, on issues such as consent for downstream research, current federal regulations for research on human participants may need to be reconsidered. Ethical guidelines are more flexible than regulations and allow consensus standards to develop on emerging issues and be applied consistently across different types of research.

In summary, iPS cells are an exciting new approach to developing pluripotent stem cell lines that are genetically identical to people with known phenotypes. While they avoid the ethical issues inherent in embryonic stem cells, they do raise some ethical concerns regarding consent for future research. Obtaining consent for fundamental downstream research with iPS cells, together with offering the options of allowing recontact by researchers and giving permission for additional sensitive types of future research, will show respect for somatic cell donors, promote public trust in stem cell research, and allow optimal use of scientific discoveries.

## Abbreviations

iPS: induced pluripotent stem
IRB: internal review board
SCRO: stem cell research oversight
